# Wolfram Syndrome 1: A Neuropsychiatric Perspective on a Rare Disease

**DOI:** 10.3390/genes15080984

**Published:** 2024-07-25

**Authors:** Valerio Caruso, Accursio Raia, Luciana Rigoli

**Affiliations:** 1 Department of Neuroscience, Psychiatric Section, Azienda Ospedaliera Universitaria Pisana (A.U.O.P.), 56126 Pisa, Italy; accursioraia1@gmail.com; 2 Department of Human Pathology of Adulthood and Childhood G. Barresi, University of Messina, 98125 Messina, Italy

**Keywords:** Wolfram syndrome 1, *WFS1* gene, neuropsychiatric disorders, new findings in Wolfram syndrome 1

## Abstract

Wolfram syndrome 1 (WS1) is an uncommon autosomal recessive neurological disorder that is characterized by diabetes insipidus, early-onset non-autoimmune diabetes mellitus, optic atrophy, and deafness (DIDMOAD). Other clinical manifestations are neuropsychiatric symptoms, urinary tract alterations, and endocrinological disorders. The rapid clinical course of WS1 results in death by the age of 30. Severe brain atrophy leads to central respiratory failure, which is the main cause of death in WS1 patients. Mutations in the *WFS1* gene, located on chromosome 4p16, account for approximately 90% of WS1 cases. The gene produces wolframin, a transmembrane glycoprotein widely distributed and highly expressed in retinal, neural, and muscular tissues. Wolframin plays a crucial role in the regulation of apoptosis, insulin signaling, and ER calcium homeostasis, as well as the ER stress response. WS1 has been designated as a neurodegenerative and neurodevelopmental disorder due to the numerous abnormalities in the ER stress-mediated system. WS1 is a devastating neurodegenerative disease that affects patients and their families. Early diagnosis and recognition of the initial clinical signs may slow the disease’s progression and improve symptomatology. Moreover, genetic counseling should be provided to the patient’s relatives to extend multidisciplinary care to their first-degree family members. Regrettably, there are currently no specific drugs for the therapy of this fatal disease. A better understanding of the etiology of WS1 will make possible the development of new therapeutic approaches that may enhance the life expectancy of patients. This review will examine the pathogenetic mechanisms, development, and progression of neuropsychiatric symptoms commonly associated with WS1. A thorough understanding of WS1’s neurophysiopathology is critical for achieving the goal of improving patients’ quality of life and life expectancy.

## 1. Introduction

Wolfram syndrome 1 (WS1) is a genetic disorder that is inherited in an autosomal recessive pattern of inheritance. WS1 is caused by mutations in the *WFS1* gene, which codes for wolframin, an endoplasmic reticulum (ER) transmembrane glycoprotein [1,2]. Homozygotes mutations in the *CISD2* gene cause Wolfram 2 syndrome (WS2), an autosomal recessive condition [3]. On chromosome 4, the *WFS1* gene is located at 4p16.1, while CISD2 is located at 4q24 [1,3,4]. In 1938, Wolfram and Wagener first described WS1 [5]. They found that all four siblings in their study, descended from consanguineous parents, developed optic atrophy (OA) and diabetes mellitus (DM). The description of more WS1 cases revealed a wide range of clinical differences, with alterations occurring in different organs [6,7]. Researchers also have referred to WS1 as DIDMOAD, as it encompasses conditions such as diabetes insipidus (DI, average age of onset: 14 years), non-autoimmune, insulin-dependent DM (6 years), OA (11 years), and deafness (D, 12.5 years) [8]. The WS1 patients are also affected by psychiatric and neurological symptoms due to extensive neurodegenerative processes in the brain [8,9]. Cognitive decline, convulsions, anxiety, and depression are very frequent neuropsychiatric symptoms [8,10,11,12]. Additional complications, including urinary system abnormalities and alterations in the endocrine system, typically complicate WS1’s clinical management [6]. The increased prevalence of renal abnormalities in WS1 has suggested the use of the acronym DIDMOADUD to define WS1 [13]. Diagnosis for WS1 occurs when the following criteria occur: (1) both major criteria (DM and OA) are present simultaneously; (2) one major criterion and two minor criteria are found; and (3) two DIDMOAD clinical features are present [13]. Many cases of WS1 initially present as insulin-dependent non-autoimmune DM, delaying immediate recognition of the disease. Studies showed that the occurrence of WS1 in patients with DM varies from 0.57% in the UK [8] to 4.8% in the Lebanese population [14]. Zmyslowska et al. observed that in populations of children with insulin-dependent DM, WS1 was identified with a delay of at least 7 years, as WS1 patients were initially misdiagnosed as having type 1 DM [15]. Moreover, Lombardo et al. reported that WS1 had a prevalence rate of 1 in 22.3 among Sicilian (Italy) patients with juvenile-onset, insulin-dependent DM who were under the age of 30 [16]. WS1 exhibits a swift progression and results in untimely mortality for affected patients, with a mean age of 30 years (25–49 years). The leading cause of mortality is respiratory failure resulting from brainstem atrophy [8]. No therapies are available for WS1. Robust clinical monitoring and supportive care may alleviate the severe and progressive symptoms of WS1. 

## 2. Epidemiology

WS1 is a very rare neurodegenerative disease. In the United Kingdom, the prevalence of WS1 is very low, at 1 in 770,000 in adults [8] and 1 in 500,000 among children [17]. In North America, it is 1 in 100,000 [18]; and in Japan, it is 1 in 710,000 [19]. Lebanese populations have a high prevalence of WS1 (1 in 68,000) [14], whereas the population from the limited region of Sicily (Italy) has a prevalence of 1 in 54,478 [16]. This is likely due to the high rate of consanguinity in these populations. There are little data about the frequency of the WS1 carriers. Barrett et al. reported that the carrier frequency in the United Kingdom was 1/354 [7]. 

## 3. *WFS1* Gene

**Figure 1:**
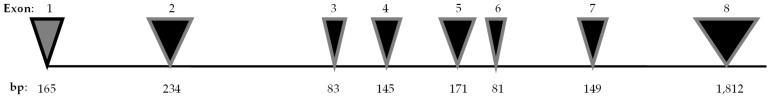
*WFS1* gene. The *WFS1* gene, which comprises eight exons, is represented with the number of base pairs (bp) for each exon [6].

In 1998, Inoue H. [1], and Strom TM. [2] mapped the human gene *WFS1* to chromosome 4p16. It spans 33.4 kilobytes and consists of eight exons. Exon 1 is non-coding, whereas the exons 2–7 are small coding regions. The largest part of the gene is exon 8 (Figure 1). 

**Figure 2:**
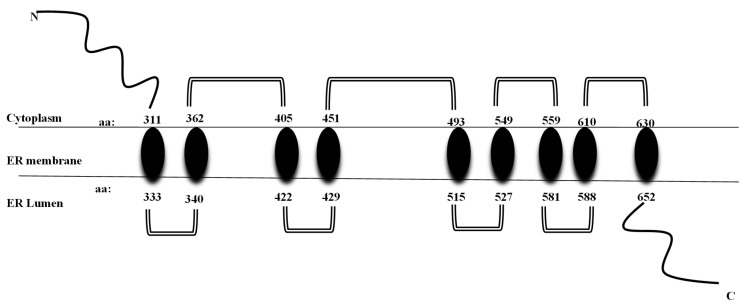
Structure of wolframin protein. The wolframin protein including cytoplasmatic, transmembrane, and endoplasmatic regions, is represented [6].

It codes for the transmembrane region and the terminal carboxy residue of wolframin, both of which are very important for the protein’s correct functioning [1]. Wolframin is a hydrophobic transmembrane glycoprotein composed of 890 amino acids. The endoplasmic reticulum (ER) contains wolframin, which has a molecular weight of 100 kilodaltons and consists of nine transmembrane domains. The secondary structure predictions show that it is composed of three distinct structural domains. These include a central hydrophobic domain consisting of 9–10 segments that cross the membrane and two hydrophilic domains situated at the N- and C-termini. The C-terminal part of wolframin is situated inside the ER lumen, while the N-terminus is located in the cytoplasm [1,20,21] (Figure 2). 

The WFS1 protein organizes into higher molecular complexes, and it interacts with other molecules to form tetramers with a molecular mass of 400 kD [21]. Wolframin is abundantly expressed in pancreatic β-cells, the heart, lungs, placenta, and brain regions such as the hippocampus, amygdala, allocortex, and olfactory bulb [21]. The protein is mostly expressed in the β-cells of the pancreas and is not found in the exocrine glands [22,23,24]. The liver and kidneys have a lower amount of wolframin [21]. Human WFS1 protein expression is low during the fetal phase (14–16 weeks of gestation) and reaches peak levels after birth [25]. Neural differentiation strongly correlates with *WFS1* mRNA expression. A reduced brain volume and specific abnormalities of the brain trunk and brain have already been highlighted in the early stages of clinical symptoms of WS1 [26]. At present, researchers have identified over 200 mutations in the *WFS1* [https://www.ncbi.nlm.nih.gov/clinvar/, accessed on 22 July 2024]. Exon 8 is the site of several mutations in the *WFS1*, including pathogenic, likely pathogenic, and uncertainly significant variants. In the other regions of *WFS1,* there are a limited number of pathogenic or potentially pathogenic variants. The mutations are mainly inactivating (nonsense or frameshift) and account for approximately 90% of WS1 cases [1,2,21,27]. The transmission of most *WFS1* mutations occurs in an autosomal recessive mode [1,2]. Nevertheless, autosomal dominant mutations have been found in WS-like diseases, such as *WFS1*-related non-syndromic low-frequency sensorineural hearing loss (LFSNHL) [28]. Due to the large number of *WFS1* mutations, the complexity of the WS1 clinical manifestations, and the relatively small number of patients, it is not easy to identify a correlation between genotype and phenotype [27,29,30]. Previous studies have shown that WS1 patients with mutations resulting in a total absence of wolframin synthesis are more susceptible to developing DM at an earlier stage compared to those with mutations resulting in a residual amount of wolframin expression [6,27] (Table 1 and Table 2).

A meta-analysis of 412 WS1 patients identified a total of 178 distinct mutations. Each patient’s WS1 phenotypic variability significantly correlated with the occurrence of different genetic variants [27]. Through the classification of mutations according to their impact on *WFS1* expression, it was found that mutations resulting in the complete absence of wolframin were associated with severe clinical manifestations of WS1. These symptoms were characterized by an earlier onset of DM and OA. WS1 patients with milder mutations had less severe clinical features [27]. In a study carried out on 44 Italian WS1 patients, the *WFS1* mutations were classified into three separate groups according to their specific effect on the expression of wolframin. Group 1 included patients with nonsense and frameshift mutations, as well as multiple amino acid insertions or deletions in both alleles of *WFS1*. This genotype resulted in a complete absence of wolframin, which in turn induced a severe clinical phenotype. Group 2 consisted of patients with biallelic missense mutations and/or single amino acid insertions. These patients showed a less pronounced deterioration of wolframin than those in group 1. Group 3 included the compound heterozygotes WS1 patients who harbored mutations that were absent in groups 1 and 2. It is intriguing that the age at which DM, D, and DI occurred varied among the three groups, while OA did not. Additionally, patients in group 1 had a shorter survival time than those in the other groups. The types of clinical features of WS1 patients were similar across the three groups [30]. There are still a lot of questions about how the symptoms of WS1 appear, but it is known that the clinical manifestations of this syndrome need *WFS1* mutations in either homozygosis or compound heterozygosis in order to show up. Numerous studies have shown that *WFS1* mutations not only alter wolframin levels but also affect RNA levels. Some patients who have frameshift mutations or compound heterozygosity for stop and missense variants show symptoms of WS1 [31]. Frameshift mutations lead to the truncation of proteins; however, this was not found in the WS1 cases that were examined. The complete absence of wolframin in WS1 patients with frameshift mutations suggests that these *WFS1* genetic variations have a significant impact. Patients with compound heterozygosity, including both stop and missense mutations, exhibited only 5% wolframin [31,32]. The inactivation of wolframin is crucial for the clinical phenotype of WS1, but the exact mechanism of inactivation remains unknown [32]. While the findings of the Koks et al. study may seem intriguing, it is crucial to point out that there are also uncommon clinical phenotypes of WS1 caused by either a single mutation or two mutations of the *WFS1*. Therefore, other genetic mechanisms may contribute to WS1’s clinical manifestation [32]. Consequently, there is a need to investigate a greater number of WS1 patients. The question of heterozygotes for *WFS1* mutations is even more complex. Generally, they do not manifest any clinical symptoms of WS1, but some studies have shown a greater susceptibility to deafness or type 2 DM [33,34]. Furthermore, heterozygotes subjects have an increased risk of suffering from psychiatric disorders with a pronounced tendency to commit suicide [35,36,37,38]. ER stress is believed to induce significant alterations in the brain areas that control emotions [39]. This could account for an increased risk of mental disorders not just in WS1 patients but also in a subset of heterozygotes with *WFS1* mutations [32]. 

## 4. Physiology and Pathophysiology of WS1 

### 4.1. Wolframin and ER Stress

Wolframin is specifically localized in the membrane of the ER, which plays a key role in cellular survival. ER is responsible for the correct folding and post-translational modification of secretory proteins, cell surface receptors, and ER transmembranes [40]. Mutations in *WFS1* cause an accumulation of misfolded proteins in the ER, resulting in ER stress. High levels of misfolded proteins stimulate the unfolded protein response (UPR) which, in turn, activates transcriptional and translational mechanisms to restore ER homeostasis. Physiological processes (post-prandial insulin biosynthesis) or pathological processes (cancer, inflammatory diseases, viral infection, gene mutations) induce a persistent state of chronic ER stress, which causes an increase in cellular apoptosis [32,40,41]. In WS1, high levels of ER stress cause pancreatic cell apoptosis and neurodegeneration [42,43]. The UPR activates three transmembrane proteins in the ER that serve as stress sensors: inositol-requiring protein 1 (IRE1), protein kinase RNA (PKR)-like ER kinase (PERK), and activating transcription factor 6 (ATF6). These transducers are crucial for enabling cellular adaptability and cell death processes. Also, a few studies have shown that immunoglobulin-binding protein (BIP) and other ER chaperones keep their lumenal domains inactive when the organism is functioning normally. BIP is synthesized to facilitate the folding of accumulated proteins, particularly when there is a high level of UPR in the ER [22]. During periods of physiological stress, the processes of IRE1 oligomerization and autophosphorylation occur. Following this, the RNase domain of IRE1 triggers the splicing of the X-binding protein 1 (XBP-1) mRNA, resulting in sXBP-1, an mRNA suitable for transcription. The activation of sXBP-1 results in XBP-1, which functions as a transcription factor. The transport of XBP-1 to the nucleus starts the expression of specific UPR target genes. This restores protein balance and starts the defense mechanisms of cells. Pathological conditions may lead to the overactivation of IRE1, resulting in apoptosis. Furthermore, IRE1 has an impact on insulin production; with high blood sugar levels, it stimulates the homeostasis of β cells and therefore enhances pro-insulin synthesis [43]. A transmembrane protein called PERK is very important for ER stress because it starts the phosphorylation of eIF2alfa, which is a eukaryotic initiation translation factor. eIF2alfa lowers the biosynthetic activity of the ER and enhances the translation of both the ATF4 transcription factor and the apoptosis antagonist transcription factors (AATF) of mRNA. ATF4 activates specific genes that are important for amino acid transport and metabolism, glutathione biosynthesis, and antioxidant responses. Moreover, pathological ER stress activates the ATF4–ATF3–CHOP complex, promoting apoptosis. Instead, the AATF factor promotes cell survival [44]. ATF6 is an important regulator of the UPR. ER stress induces the dissociation of BIP, allowing ATF6 to translocate to the Golgi apparatus. Within the Golgi apparatus, some proteases are responsible for the cleavage of ATF6, leading to the subsequent production of a transcription factor that is active in the cytoplasm. When activated, ATF6 migrates to the nucleus, increasing protein folding, processing, and degradation activity by upregulating ER transcriptional homeostatic factors. Furthermore, studies have demonstrated that ATF6 plays a regulatory role in the process of lipid biosynthesis [43,45]. During physiological ER stress, *WFS1* has a negative regulatory function in the complicated UPR process. *WFS1* inhibits ATF6, decreases the activation of the ER stress response element (ERSE), and promotes a stable state of E3 ubiquitin ligase HRD1, a protein involved in the degradation of HMG-CoA reductase. Consequently, *WFS1* suppresses stress signals. In WS1, on the other hand, the excessive ATF6 activation increases the production of apoptosis-related genes like CHOP, ATF4, BIP, and sXBP1 while decreasing the expression of insulin-related genes [40]. It is believed that wolframin deficiency causes an elevated UPR, which intensifies neurodegenerative damage by triggering the ER stress mechanism. Recent neuroimaging investigations have emphasized that WS1 is associated with a significant disruption in early brain development, specifically affecting the myelinization of the white matter. Some genetic myelin disorders, such as Pelizaeus–Merzbacher’s disease and Vanishing White Matter disease, have shown similar alterations. Thus, the occurrence of demyelination in response to ER stress indicates that WS1 should be considered a disturbance in neurodevelopment [46]. 

### 4.2. Wolframin, Calcium, and Mitochondria

The release and uptake of calcium ions in the ER also influences cellular apoptosis. Researchers have discovered that wolframin functions as a calmodulin (CaM) that interacts with many cellular proteins and controls the Ca^2+^ signaling pathways linked to apoptosis [41]. Because increased ER stress levels lead to alterations in mitochondrial function, many researchers have suggested that WS1 may be defined as a mitochondrial disease [47]. Numerous studies have provided evidence for a correlation between ER stress, high cytosolic Ca^2+^ levels, impaired mitochondrial dynamics, and inhibited neuronal development in *WFS1*-deficient neurons [47,48]. In healthy cells, *WFS1* links to neuronal calcium sensor 1 (NCS1) and the inositol 1,4,5-trisphosphate receptor (IP3R) to promote the transfer of Ca^2+^ between the ER and the mitochondria. *WFS1*-deficient cells exhibit a significant reduction in NCS1 levels. Consequently, there is a loss of interactions between the ER and mitochondria, as well as a decrease in the transfer of calcium ions (Ca^2+^) [47]. Therefore, there is a strong link between ER stress, modifications in cytosolic calcium levels, changes in mitochondrial dynamics, and developmental delay in *WFS1*-deficient neuronal cells [49]. The modifications of mitochondria-associated ER membranes (MAMs) substantially influence this intricate pathogenic mechanism [50]. MAMs, which are dynamic domains of interaction between the mitochondria and ER, are the specific sites of several proteins that are implicated in UPR. These proteins are responsible for stabilizing the structure of MAMs and for enabling the functional interaction between the ER and mitochondria. MAMs are responsible for the main transport of Ca^2+^ between the ER and mitochondria through the inositol 1,4,5-triphosphate receptor (IP3R) calcium channel [50,51]. The ER stress cascade inhibits the function of the IP3R calcium channel, leading to a subsequent disruption in the homeostasis of cytosolic Ca^2+^ levels. This altered pathway leads to alterations in mitochondrial dynamics, including inhibited mitochondrial fusions, abnormal mitochondrial trafficking, and enhanced mitophagy. Alterations in mitochondria lead to decreased ATP levels, which in turn affect the development of neurons. The analysis of the proteins involved in mitochondrial function revealed a downregulation of the subunits of the respiratory chain complexes, an upregulation of the proteins involved in the Krebs cycle, and glycolysis mechanisms in WS neural stem cells (NSC) [51]. There are similarities between the neurological and psychiatric symptoms of WS1 and those seen in mitochondrial diseases, which lead some authors to hypothesize that mitochondria may help explain the symptoms of WS1 [52]. ER stress can cause serious changes in mitochondrial dynamics that can lead to a “mitochondrial phenotype” in WS1 patients [47]. Zmyslowska et al. used human WS1 cells as a model to study the role of mitochondria in WS1. Initially, the researchers transformed skin fibroblasts into induced pluripotent stem cells (iPS), which subsequently underwent further differentiation into NSC. They were then exposed to ER stress. In WS1 NSC, an evaluation of the proteins associated with mitochondrial activity revealed a downregulation of the subunits of the respiratory chain complexes, an upregulation of the proteins involved in the Krebs cycle, and mechanisms of glycolysis. In contrast, the control cells did not exhibit similar changes. Furthermore, it was suggested that alterations in the structure and function of the mitochondria are crucial in the development of WS1 [51]. Wolframin directly influences the relationship between mitochondria and the ER, which is crucial for cellular metabolism and survival. The significant influence on cellular physiology explains the intricate nature of WS1, a disease that impacts several systems. Nevertheless, it is crucial to point out that *WFS1* mutations do not directly affect the morphology and functions of the mitochondria [53]. The influence of *WFS1* on the mitochondria is mediated by the interactions between IP3R, voltage-dependent anion channel 1 (VDAC1), and glucose-regulated protein 75 (GRP75), also known as heat shock protein family (Hsp70) member 9 (HSPA9). Thus, wolframin has a strong influence on both vesicular traffic and the ER-mitochondrial relationship. The connection between *WFS1* and NCS1 is very important for this complicated link because it may explain the alterations found in the respiratory chain in WS1 patients and mutant mouse muscles [53]. Koks et al. have also shown that the silencing of the *WFS1* gene in HEK cells alters TOMM20, a protein associated with the ER-mitochondria transport [54,55]. Thus, the mutations in the *WFS1* gene do not have a direct influence on mitochondria. The impact on mitochondrial function occurs via a complicated interactions between multiple protein complexes, and the communication between the ER and mitochondria is critical for the disease’s underlying mechanisms. 

### 4.3. Wolframin and Neurodevelopment

Wolframin plays a crucial role in brain development; however, the specific processes through which it functions remain incompletely understood. The onset of wolframin expression in the brain occurs at an early stage. In fact, some authors found clinical manifestations of WS1 as early as the intrauterine and early postnatal years [56,57]. From 8 to 15 years of age, the human brain exhibits the highest expression of wolframin, indicating that *WFS1* is more active during early brain development than in maturity. Researchers have found that between late childhood and early adolescence, there is a phase of active myelination in the neurodevelopmental process that varies across the different regions of the brain, such as the cortex and subcortex [58]. Other studies have shown that myelination undergoes dynamic changes during pre-adolescent and adolescent periods, particularly in the formation of the hippocampus [46]. Lugar et al. have found that myelination increases in the motor and cingulate cortices during adolescence [59]. Conversely, other studies have described myelination processes in the frontopolar and visual neocortex during development, but not in the motor and somatosensory cortices [60]. Studies showed a significant increase in wolframin mRNA expression in mice’s brains during the period from adolescence to early adulthood. In addition, the amount of *Wfs1* mRNA in the supraoptic nucleus and magnocellular nucleus of mice remained rather stable during their growth, but it declined after birth [61,62]. Rats had higher levels of *Wfs1* mRNA and wolframin in specific areas of their limbic system, like the amygdaloid area, the CA1 region of the hippocampus, the olfactory tubercles, basal ganglia, several brainstem nuclei, and the surface layer of the piriform allocortex [58]. On the other hand, the expression patterns of *WFS1* vary depending on the phases of brain development and the different areas of the brain [59]. It has also been suggested that *WFS1* plays a key functional role in the development and function of neuronal cells located in the hypothalamic nuclei, the auditory system, and the cerebellum. Interestingly, all areas of the brain express *WFS1*. However, in WS1, neuronal loss is prevalent exclusively in specific brain regions, including the cerebellum, the optical pathway, and the brain trunk. Some studies have shown that certain types of neurons are less susceptible to *WFS1* mutations than others. In some brain regions, unknown proteins or pathways compensate for wolframin deficiency, while other neurons are more susceptible [62,63]. Understanding the various mechanisms of wolframin expression in the brain could provide insight into the various neuropsychiatric manifestations of WS1. Tekko et al. studied the initial pattern of *Wfs1* expression in the mouse forebrain using mRNA in situ hybridization. The expression of the synaptophysin (Syp1) gene, which codes for a protein found in synaptic vesicles, was assessed as a marker of neuronal development and synaptic connections. It was found that the expression of *Wfs1* started during the later stages of embryonic development in the dorsal striatum and amygdala, and then increased after birth. Syp1 expression preceded *Wfs1* and significantly increased during the onset and maturation phases of *Wfs1* expression, indicating a link between neuronal activity and *Wfs1* expression. Only after each brain structure had fully developed did the *Wfs1* expression start to manifest, reaching the adult pattern three weeks after birth. *Wfs1* expression was either absent or very weak during the early phases of brain development [58]. Thus, some authors suggested that *WFS1* could regulate independently during the postnatal period, potentially protecting the brain against neurodegeneration. In HEK cells, silencing *WFS1* activates a complex molecular network known as the “protein trafficking, cell morphology, cellular function, and maintenance network”. It has been found that some genes (ADAM 19, TOMM20, and EPAS1) and proteins (transthyretin and heat shock protein 8) could work together to damage neurons by changing the UPR or mitochondrial systems. In the adult central nervous system, Wfs1 expression is abundant in the amygdala, striatum, and hippocampus, all of which are significantly involved in the organism’s behavioral adaptation [55,58]. 

### 4.4. WFS1: Altered Neurodevelopment and Neurodegeneration

The tissue volume greatly influences normal neurodevelopment. So, from childhood to adulthood, there is a gradual and constant increase in white matter, probably due to axon myelination. Instead, the volume of gray matter reaches its peak at an early age and subsequently decreases during development due to synapse elimination and dendritic pruning [46,64]. These processes occur differently depending on the various areas of the brain, with higher-order cortical regions that develop later than the primary sensory areas [65]. Significant disruptions in the pathways that characterize normal neurodevelopment distinguish WS1, according to numerous studies [46,64,65]. Lugar et al. found that WS1 patients had brain abnormalities, including a smaller intracranial volume and alterations in white matter integrity (brainstem and ventral pons), whereas controls, matched for age and sex, had a significant increase in white matter volume in the thalamus and cerebellar cortex [66]. These abnormalities were more noticeable in younger patients. Therefore, the early phases of WS1 were characterized by stalled white matter development and additional degenerative processes in both white and gray matter. The late stages of WS1 exhibited widespread cerebral atrophy. The etiology and specific features of these modifications are still not understood.

Hypo- and inhibited myelination characterize the brain anomalies in the early stages of WS1, while degenerative processes affecting the axons and neurons likely cause the later stages. So, many studies have shown that the neurological symptoms of WS1 are caused by different anomalies in specific areas of the brain, such as neurodegeneration and neurodevelopmental processes [66]. It has been suggested that WS1 patients experience impairments in movement, sensory, and other functions, such as respiration, due to alterations in white matter loss in the brainstem and pons. Previous studies have associated abnormalities in the ventral pons volume to impaired renal function and alterations in the cerebellum to gait and balance complications [26,66] Neuropathological studies have shown a lack of neurons, myelin, and myelinated axons in the brains of WS1 patients [46]. However, the specific moment during which these alterations occur is still uncertain. Some studies have found a link between WS1 and congenital neurological disorders, such as orbital bone and eye globe hypoplasia [67], as well as neural tube defects like spina bifida [68], and most likely microcephaly [26]. These observations reinforce the hypothesis that WS1 is a neurodevelopmental disease that manifests neurodegenerative characteristics in its later stages. 

### 4.5. Oligodendrocytes

Wolframin is crucial for the early stages of brain development, being involved in processes such as neurogenesis, neuronal migration, and myelination [47,60,66]. In fact, the UPR, which is regulated by the expression of *WFS1*, is activated during the physiological process of myelinization of the brain. Oligodendrocytes, the myelinating cells of the central nervous system (CNS), have a considerable impact on the development and maintenance of axonal integrity [69,70]. Because they make a lot of plasma membranes and transmembrane proteins during the myelination process, oligodendrocytes are very sensitive to changes in the secretory pathway. Studies have already shown that the activation of UPR in oligodendrocytes during active myelinization leads to cell death and apoptosis [47]. It has been proposed that a wolframin deficiency in actively myelinating oligodendrocytes triggers the UPR, leading to aberrant myelination and oligodendrocyte death. Many studies have suggested that *WFS1* is essential for the optimal functioning of oligodendrocytes. However, we still lack complete clarity on the precise roles of *WFS1* and wolframin in oligodendrocytes. Understanding the impact of wolfram deficiency on neuronal cell differentiation and maturation is important, as the expression of the *WFS1* gene varies during intrauterine life and then during the various phases of postnatal life. Wolfram syndrome’s initial stages are characterized by abnormal myelin development, as shown by some studies [60]. To elucidate these observations, two hypotheses have been proposed: firstly, the function of oligodendrocytes is altered by wolfram deficiency, which disrupts myelin development. Secondly, ER stress causes myelin degeneration, which in turn induces cellular apoptosis [47,60]. Pelizaeus–Merzbacher (PMD) and Vanishing White Matter disease (VWMD) show analogous mechanisms [60,71]. 

### 4.6. Histopathological Alterations in Wolfram Syndrome 1

Neuropathological studies are crucial in identifying brain regions and structures involved in WS1. Postmortem brain histopathological case studies have shown that sensory pathways, the brainstem, cerebellum, and hypothalamus are the most affected brain regions, varying in age, cause of death, and examined tissues. In the visual system, the optic nerve exhibits significant degeneration, including a loss of retinal ganglion neurons and myelinated axons in the visual pathways. However, the visual cortex appears relatively unaffected [72,73]. The auditory pathways are characterized by the loss of the organ of Corti in the basal turns of the cochlea, as well as the loss of fibers in the cochlear nerve and neurons in the cochlear nuclei and inferior colliculus [72]. The olfactory pathway exhibits atrophy in both the olfactory bulb and tract [74], whereas the brainstem and cerebellum show a decrease in volume. The histopathological alterations include modest neuronal loss and gliosis in almost all brain nuclei, including the pontine nuclei, lower olivary nucleus, vestibular nucleus medial, medullary and pontine reticular form, hazy dorsal nuclei, and ambiguous nuclei [72,73,74]. WS1 was shown in some cases by a loss of neurons in the dentate nuclei and a rise in Purkinje cells in the cerebellum. In some WS1 cases, the hypothalamus exhibited gliosis and a severe loss of magnocellular neurons in the supraoptic and paraventricular nuclei [72,73,74]. Conversely, the thalamus and other brain structures might suffer less damage. Some histopathological studies found that the anterior and dorsomedial nuclei exhibited relatively small neuronal loss and gliosis. Mild axon damage in the calcarine cortex, mild motor neuron loss and gliosis in the spinal cord, and pigment loss, neuronal loss, and gliosis in the substantia nigra are all less common features [74]. These data have suggested that WS1 could be characterized by two separate histopathological pathways. Neuronal loss and gliosis have been found in the subcortical and limited cortical gray matter. In contrast, areas of demyelination and axonal degeneration have been detected in many parts of the white matter, such as the optic radiation, pontocerebellar and corticopontine tracts, hippocampus fornices, and the deep cerebral white matter [72,73,74]. These observations have indicated that neuronal loss is a critical factor in WS1 development that likely operates apart from other pathogenic pathways. Based on histopathological research, we can deduce that the neuropathological changes in WS1 range from a slight decrease in the number of axons and myelin to a larger loss of myelin, axons, and neurons, as in the latter phases of the disease [60,67]. 

## 5. *WFS1* and Neuropsychiatric Disorders

WS1 patients often exhibit neuropsychiatric complications, which are considered the third clinical manifestation of WS1 after DM and OA. Sixty-two percent of patients exhibit neurological symptoms [8]. Conversely, a separate study found a significantly higher occurrence of neurological diseases, with percentages reaching as much as 70% [10]. Normally, the onset of neurological abnormalities occurs at an average age of 16 years (range 5–44 years old), but in some cases, the onset is earlier [10]. Indeed, many studies have shown the occurrence of subclinical neurological signs in the first phases of the disease, namely during late puberty. A study by Heredia et al. showed that neurological symptoms appeared at 10–30 years of age with a median of 23 years and two peaks—one at 13 and the other at 30 years of age [27]. In 54% of WS1 patients, magnetic resonance imaging (MRI) revealed several cerebral abnormalities. However, patient evaluations often occur during the advanced stages of the illness, leaving the precise brain abnormalities that occur in the early phases of the disease unclear [47,72,75]. Brain scans showed that WS1 patients had a severe atrophy of the brainstem, and both gray and white matter in the cerebellum, the posterior area of the cerebellum, and the optic nerve. Occasionally, the radiological scans showed a slight level of brain atrophy in children with WS1 [47]. Regrettably, there is often a divergence between the radiographic and neurological signs. Sometimes, a patient with significant brain alterations has only mild symptoms. Abnormalities in the cerebellum cause trunk ataxia, the most common neurological symptom of WS1 (45%). Some studies found that fifteen out of the forty-five patients with WS1 had trunk or gait ataxia [7,8,10]. Therefore, it is recommended that neurological counseling be offered to WS1 patients at least once or twice a year [10,75]. Brain stem atrophy frequently leads to respiratory failure or dysphagia [8,10]. In these cases, polysomnography and a nocturnal oximetry test are required. Occasionally, a tracheostomy is necessary. Aspiration pneumonia may be caused by dysphagia, which can be relieved with swallowing therapy. Esophageal dilatation and esophagomyotomy can be beneficial in particular cases. Peripheral neuropathy (39%), cognitive impairment (32%), epilepsy (26%), and dysarthria, dysphagia, and nystagmus (10%) are the other neurological disorders caused by WS1 [6,10]. Orthostatic hypotension, anhidrosis, hypohidrosis, or hyperhidrosis, constipation, gastroparesis, hypothermia, or hyperpyrexia are all common neurological symptoms [6,10]. WS1 patients also report a history of migraines [7]. Urinary incontinence, neurogenic bladder with hydroureter, and frequent infections may also result from neurological abnormalities affecting the urinary tract. For patients who exhibit these signs, a urodynamic examination is required, as it may identify bladder atony or incomplete bladder evacuation [7,8,10]. 

### Psychiatric Disorders

Numerous WS1 patients exhibit various psychiatric symptoms in their early adulthood, with an average onset age of 20.5 years [7,8,31]. Nevertheless, many authors have suggested that psychiatric signs may be absent during the first phases of WS1 but may manifest at later stages [36]. The variability in the age at which psychiatric disorders appear may be linked to WS1’s continued neurodegenerative processes. As a result, cognitive and psychological symptoms frequently become apparent during the later phases of WS1 disease. The cause of these psychiatric disorders is still unknown, as it is unclear if these symptoms arise only from stress related to a chronic disease, genetic changes in the *WFS1* gene, or a combination of both causes. In WS1 patients, a history of psychiatric symptoms such as severe depression with suicide attempts, psychosis, sleep irregularities, verbal impulsivity, and aggression could aggravate the overall clinical picture [6,8,36]. Some authors found that 77% of WS1 patients had anxiety as their prevalent symptom. Specifically, WS1 patients have an increased risk of developing anxiety and obsessive-compulsive spectrum disorders [76]. A study showed that 60% of patients diagnosed with WS1 had a history of severe psychiatric disorders, including depression, psychosis, disorientation, deficits in memory, dementia, irritability, frustration, and impulsive aggression. Twenty-five percent of these patients had a “very severe” condition, with 12 of them needing hospitalization and 11 attempting suicide. The age range for the first suicide attempt or hospitalization was between 15 and 32 years [76]. WS1 patients often have a positive response to conventional therapies. The management and follow-up of WS1 patients who have made suicide attempts requires urgent multidisciplinary care. Previous studies have shown the potential use of smell- and sleep-related symptoms as useful markers for monitoring WS1 patients with psychiatric symptoms [76].

In general, patients with WS1 do not exhibit signs of cognitive decline. Cognitive impairment was the third symptom (32%) in a group of 59 WS1 patients. This occurred after cerebellar ataxia and peripheral neuropathy [10]. 

Although heterozygous carriers do not exhibit any symptoms, they are at significant risk of developing a spectrum of psychiatric symptoms associated with the *WFS1* mutations [10,76,77]. Numerous studies have established a link between heterozygous *WFS1* variants and deafness, as well as an increased risk of developing type 2 DM [78]. *WFS1* heterozygosity has also been associated with metabolic diseases, psychiatric disorders, and even suicidal ideation [79]. Swift et al. found that 10 of 11 hospitalized relatives of WS1 patients were heterozygotes for *WFS1* mutations. These findings revealed that *WFS1* heterozygotes had a 26-fold increased incidence of psychiatric hospitalization compared to non-heterozygotes [80].

Recent studies have suggested that *WFS1* may be a promising target for psychiatric disease research because of the high prevalence of psychiatric disorders among WS1 patients and their families. However, understanding the pathophysiology of psychiatric disorders in WS1 patients presents several difficulties. 

Because *WFS1* is highly expressed in the limbic system, which includes the amygdala, the hippocampal region, the olfactory tubercles, and the top layer of the piriform allocortex, it has been proposed that alterations in these brain regions cause psychiatric disorders in WS1 patients [6,81,82]. Some studies showed that *Wfs1*-deficient mice manifested behavioral traits similar to those observed in WS1 patients, such as anxiety, depression, and post-traumatic stress disorder [81,82]. We still do not fully understand the exact mechanism that triggers psychiatric symptoms, but it seems that ER stress plays a key role because it could modify the regulatory brain centers of emotions [83].

Sleep difficulties are psychological disorders that could characterize WS1’s clinical picture. When compared to healthy individuals, WS1 patients and their parents may have greater sleep-related symptoms, such as snoring, heavy breathing, bed wetting, and excessive tiredness. Furthermore, a large number of WS1 patients in adolescence and adulthood exhibit hypersomnolence disorders [84]. There is not a clear link between WS1 and sleep disorders because it is not known if these sleep disorders are mainly caused by WS1 or by other chronic diseases like type 1 diabetes or diabetes insipidus, which can increase the frequency of nocturnal urine and disrupt sleep patterns [84]. It is critical to understand wolframin’s role in sleep regulation since heterozygous *WFS1* mutations affect around 1% of the population and have a significant potential influence on psychiatric disorders. Many studies found a substantial incidence of respiratory abnormalities during sleep, notably obstructive sleep apnea (OSA), in WS1 patients. Adults and children with WS1 had significantly higher OSA rates than the general population (29.4% vs. 2–7% for adults, and 100% vs. 1–5% for children). Studies have suggested that the high prevalence of OSA in WS1 patients may influence WS1 progression [82,84,85]. Indeed, studies have found a correlation between a high apnea–hypopnea index (AHI) and increased disease severity [84]. Sleep disruption in WS1 may be associated with localized neuropathology, such as reduced brainstem and cerebellar volumes [26]. Studies have suggested that alterations in ER-mediated calcium homeostasis, which regulates the neuronal activity and neurotransmitter release of molecules such as dopamine, make WS1 patients more vulnerable to sleep disorders [86,87].

Despite extensive research, the role of *WFS1* in the onset of psychiatric symptoms in Wolfram syndrome 1 (WS1) remains poorly understood. To date, specific *WFS1* mutations have not been definitively linked to psychiatric disorders in WS1 patients. This lack of a clear genotype–phenotype correlation poses significant challenges in understanding the neuropsychiatric aspects of the disease.

In the literature, there are reports of WS1 patients with psychiatric symptoms who harbor mutations in the *WFS1* gene (Table 3). 

However, these cases do not establish a consistent pattern or a direct causal relationship between specific *WFS1* mutations and psychiatric manifestations. The variability in psychiatric symptoms among WS1 patients further complicates the identification of genetic factors directly responsible for these symptoms.

One possible explanation for this complexity is the multifactorial nature of psychiatric disorders, which may result from a combination of genetic predispositions, environmental influences, and other biological factors. The *WFS1* gene is known to be involved in various cellular processes, including ER stress response and calcium homeostasis, which are crucial for neuronal function and survival. Disruptions in these processes due to *WFS1* mutations could potentially contribute to the neuropsychiatric symptoms observed in WS1 patients, but the exact mechanisms remain elusive.

Moreover, the expression of *WFS1* in brain regions associated with emotional regulation, such as the amygdala and hippocampus, suggests a potential link to psychiatric disorders. Nonetheless, the current evidence is insufficient to draw definitive conclusions about the specific mutations or pathways involved.

**Figure 3:**
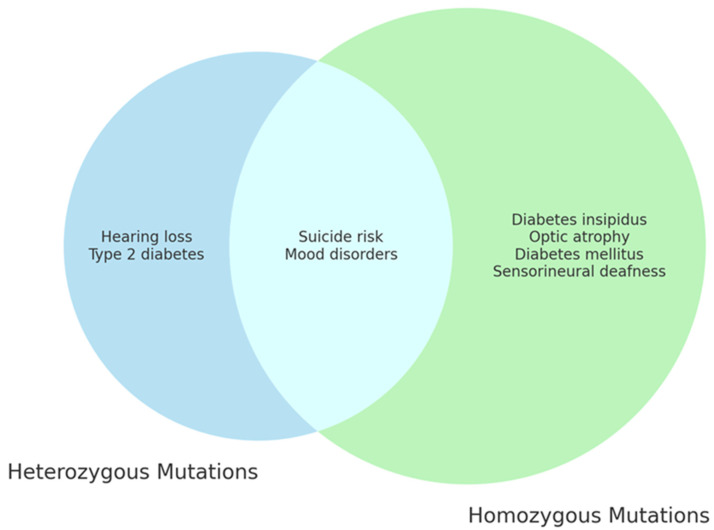
*WFS1* mutations and clinical features of WS1. The diagram highlights the distinct and overlapping clinical features resulting from different genetic mutations of the *WFS1* gene, showing the complexity and the variety of clinical manifestations associated with Wolfram syndrome.

Further research is needed to elucidate the role of *WFS1* in the development of psychiatric symptoms in WS1 (Figure 3). 

This includes detailed genetic studies, functional analyses of different *WFS1* mutations, and comprehensive clinical evaluations of WS1 patients. Understanding these mechanisms may eventually lead to better management and therapeutic strategies for the neuropsychiatric aspects of WS1.

## 6. Conclusions

A multidisciplinary approach is required to treat the severe clinical manifestations of WS1, a rare genetic syndrome that is characterized by severe neurodegeneration and premature death. An early diagnosis of WS1 might improve the patient’s clinical condition. As WS1 is a genetic disorder, it is imperative that the affected patients’ relatives receive genetic counseling. The pathogenesis of this deadly disease is, unfortunately, not completely understood. New research indicates that ER Ca^2+^ dyshomeostasis, UPR dysregulation, Ca^2+^, mitochondrial dyshomeostasis, mitocondrium dysfunction, mitophagia, and cytotoxicity are all important factors in the onset of WS1. 

WS1 is considered an ER stress pathology, which is the main factor in the significant clinical consequences associated with this disease.

Furthermore, a strong relationship has been established between ER stress dysregulation and a variety of pathologies, including specific metabolic disorders (type 1 and type 2 diabetes), neurodegenerative diseases, atherosclerosis, inflammatory diseases, and even cancer. Therefore, it has been proposed that the understanding of the connections between ER stress, alterations in the Ca^2+^ cytosol, mitochondrial dynamics, and neurodevelopment, as shown in WS1, could also contribute to the comprehension of the pathogenesis of other diseases.

## Figures and Tables

**Table 1:** Genotypic classification of *WFS1* mutations.

Groups of Mutations	Localization of Mutations	Type of Mutations	Type of Alterations of Wolframin
type 1	before exon 8	nonsense and frameshift	complete deletion
type 2	aa 1-aa 670 aa 701-aa 890	missense nonsense	complete degradation
type 3	after exon 8 and before aa700	nonsense	expression of a defective or shorter protein
	after exon 8	frameshift	
	aa 671-aa 700	missense	

de Heredia et al., 2013 [27].

**Table 2:** Functional alterations of wolframin according to type of WFS1 mutations.

Class		Functional Alterations
A	A1	wolframin depletion due to *WFS1* mRNA degradation
	A2	wolframin depletion due to mRNA and protein degradation
	A3	wolframin depletion due to protein degradation
B		reduced expression of a defective wolframin
C		expression of a defective wolframin

de Heredia et al., 2013 [27].

**Table 3:** Some *WFS1* mutations in WS1 patients exhibiting neuropsychiatric symptoms.

WFS1 Mutation	Type of Mutation	Exonic Localization	Wolframin’ Sites	Reference
p.F883X	Nonsense	Exon 8	C-terminal transmembrane domain	Inoue et al., 1998 [1]
c.1628G>T (G702S)	Missense	Exon 6	Transmembrane domain	Aloi et al., 2012 [88]
c.C529T	Missense	Exon 2	N-terminal domain	Du et al., 2018 [89]
c.C529A	Missense	Exon 2	N-terminal domain	Hofmann et al., 2003 [21]
c.C1885T	Missense	Exon 6	Transmembrane domain	Du et al., 2020 [89]
c.2050G>A	Missense	Exon 8	C-terminal transmembrane domain	Xavier et al., 2021 [90]
c.376G>A	Missense	Exon 2	N-terminal domain	Riachi et al., 2019 [91]
c.1480G>A	Missense	Exon 2	N-terminal domain	Hofmann et al., 2018 [21]
p.Arg558Cys	Missense	Exon 6	Transmembrane domain	Urano et al., 2022 [92]
p.Glu809Lys (c.2425G>A)	Missense	Exon 6	Transmembrane domain	Chaussenot et al., 2011 [10]
p.Glu864Lys (E864K)	Missense	Exon 8	C-terminal transmembrane domain	Inoue et al., 1998 [1]
p.Gln857Arg (c.2569A>G)	Missense	Exon 8	C-terminal transmembrane domain	Cryns et al., 2003 [36]
p.Pro724Leu	Missense	Exon 8	C-terminal transmembrane domain	Inoue et al., 1998 [1]

This table provides details on some WFS1 gene mutations, including the type of mutation, exonic localization, affected region of the wolframin protein, and the corresponding bibliographic references. These mutations are found in some patients with Wolfram syndrome 1 (WS1) who exhibit neuropsychiatric symptoms.
